# Supplementary figures and images for: Assessing the Genomics Structure of Dorper and White Dorper Variants, and Dorper Populations in South Africa and Hungary
Source: Biology (Basel). 2023 Feb 28;12(3):386. doi: 10.3390/biology12030386 (PMC10045292; doi:10.3390/biology12030386)

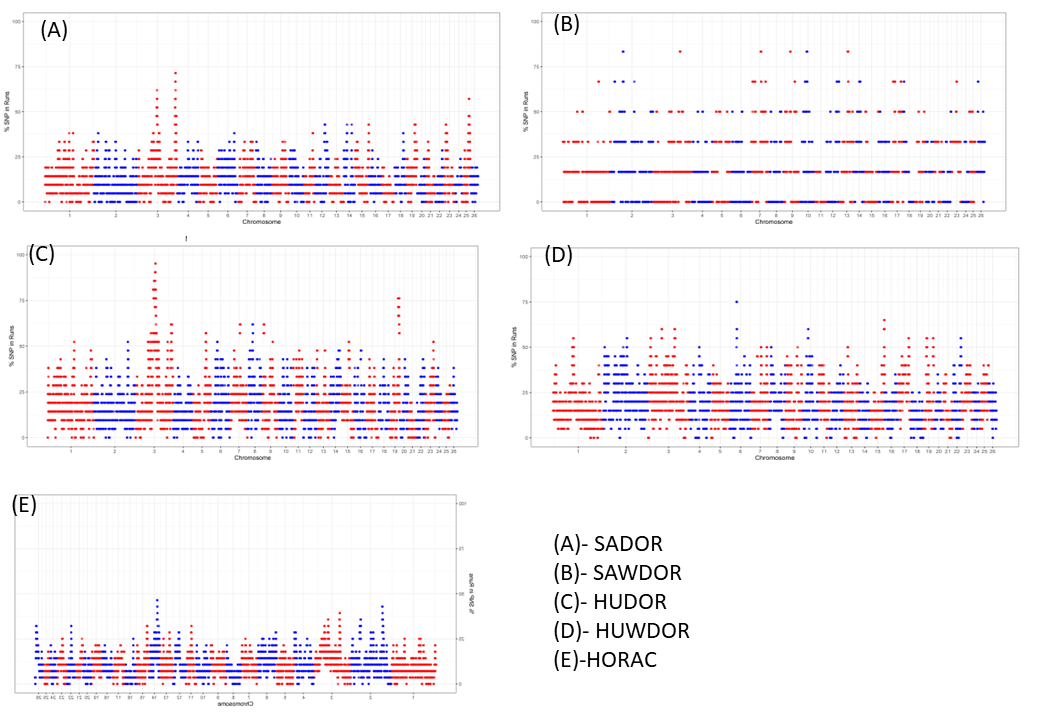

Supplement: Supplementary file 1 [file biology-12-00386-s001.zip › Figure S1.tif]
