# Supplementary material for: Assessing the Genomics Structure of Dorper and White Dorper Variants, and Dorper Populations in South Africa and Hungary
Source: Biology (Basel). 2023 Feb 28;12(3):386. doi: 10.3390/biology12030386 (PMC10045292; doi:10.3390/biology12030386)
Supplement: Supplementary file 3 [file biology-12-00386-s001.zip › Table S1.odt]

Table S1: Genes that may be under selection across all populations studied.

|  | Gene name | Gene symbol | Chromosome | Ensemble ID | Start position | End position | Possible function |
| --- | --- | --- | --- | --- | --- | --- | --- |
| HUDOR | Uncoupling Protein 2 | UCP2 | 1 | ENSOARG00000001385 | 155066999 | 155067805 | control of body temperature and regulation of energy balance. |
| Signal Transducer And Activator Of Transcription 1 | STAT1 | 15 | ENSOARG00000008561 | 51596036 | 51598758 | Immunity during early pregnancy |
| Interleukin-7 | IL7 | 2 | ENSOARG00000013903 | 192154022 | 192352057 | Immunity |
| Fatty Acid Binding Protein 5 | FABP5 | 9 | ENSOARG00000007883 | 54817997 | 54825977 | fatty acid uptake, transport, and metabolism. |
| Fatty Acid Binding Protein 4 | FABP4 | 9 | ENSOARG00000008976 | 57354087 | 57359292 | fatty acid uptake, transport, and metabolism |
| HUWDOR | Keratin associated protein 7 | KAP7 | 1 | ENSOARG00000007941 | 122988317 | 122988574 | Wool production |
| Guanylate Cyclase Activator 2A | GUCA2A | 1 | ENSOARG00000020385 | 16472798 | 16474553 | immunity |
| Interleukin-2 | IL2 | 17 | ENSOARG00000000044 | 34935045 | 34939550 | immunity |
| Fibroblast Growth Factor 2 | FGF2 | 17 | ENSOARG00000017222 | 34113507 | 34661626 | immunity |
| Interleukin 21 | IL21 | 17 | ENSOARG00000017881 | 34778947 | 34786075 | immunity |
| Nuclear Factor, Interleukin 3 Regulated | NFIL3 | 2 | ENSOARG00000003352 | 26301791 | 26303179 | immunity |
| Cytochrome P450 Family 17 Subfamily A Member 1 | CYP17A1 | 22 | ENSOARG00000002137 | 22557939 | 22564240 | metabolism |
| Plasminogen Activator, Urokinase | PLAU | 25 | ENSOARG00000008473 | 29676048 | 29685840 | immunity |
| Lactalbumin Alpha | LALBA | 3 | ENSOARG00000019223 | 137390403 | 137392415 | milk quality |
| Neuropeptide VF Precursor | NPVF | 4 | ENSOARG00000010230 | 70625131 | 70628520 | Regulates sleep |
| Albumin | ALB | 6 | ENSOARG00000013782 | 88136611 | 88159187 | regulation of blood pressure |
| C-X-C Motif Chemokine Ligand 8 | CXCL8 | 6 | ENSOARG00000014496 | 88474889 | 88477180 | growth and immunity |
| C-X-C Motif Chemokine Ligand 1 | CXCL1 | 6 | ENSOARG00000014775 | 88610487 | 88611559 | growth and immunity |
| SADOR | MX Dynamin Like GTPase 2 | MX2 | 1 | ENSOARG00000010231 | 259688071 | 259716801 | reproduction and immunity |
| MX Dynamin Like GTPase 1 | MX1 | 1 | ENSOARG00000010283 | 259737321 | 259762804 | reproduction and immunity |
| Oviductal Glycoprotein 1 | OVGP1 | 1 | ENSOARG00000019599 | 87968079 | 87982027 | reproduction |
| Adenosine A3 Receptor | ADORA3 | 1 | ENSOARG00000019630 | 88054043 | 88056874 | Immunity |
| Endoplasmic Reticulum Protein 29 | ERP29 | 17 | ENSOARG00000010504 | 61783505 | 61790600 | digestion |
| PC4 And SRSF1 Interacting Protein 1 | PSIP1 | 2 | ENSOARG00000014002 | 83388239 | 83424864 | Electron transport, Respiratory chain, Transport |
|  | NADH:Ubiquinone Oxidoreductase Subunit B8 | NDUFB8 | 22 | ENSOARG00000015378 | 20557569 | 20562826 | Respiration |
| Interleukin 21 Receptor | IL21R | 24 | ENSOARG00000018894 | 24987772 | 25002160 | immunity |
| Growth Hormone Releasing Hormone Receptor | GHRHR | 4 | ENSOARG00000007636 | 65350354 | 65364813 | growth |
| Aquaporin 1 | AQP1 | 4 | ENSOARG00000007775 | 65387133 | 65430981 | Homeostasis |
| Cortactin Binding Protein 2 | CTTNBP2 | 4 | ENSOARG00000010183 | 50432535 | 50603933 | Nervous system |
| Myozenin 3 | MYOZ3 | 5 | ENSOARG00000007579 | 59545166 | 59557329 | Muscle development |
| SAWDOR | Fc Gamma Receptor IIIa | FCGR3A | 1 | ENSOARG00000010070 | 111039478 | 111049403 | immunity |
| Fc Gamma Receptor II a | FCGR2B | 1 | ENSOARG00000010094 | 111115681 | 111132021 | immunity |
| Superoxide Dismutase 1 | SOD1 | 1 | ENSOARG00000007880 | 122094296 | 122094754 | immunity |
| BTG Anti-Proliferation Factor 3 | BTG3 | 1 | ENSOARG00000015790 | 137814444 | 137830717 | immunity |
| Ubiquilin 1 | UBQLN1 | 2 | ENSOARG00000009010 | 35845653 | 35897664 | skeletal muscle growth |
| Proline Rich 15 | PRR15 | 4 | ENSOARG00000008451 | 66566695 | 66567075 | Reproduction |
| Interleukin-7 | IL7 | 9 | ENSOARG00000007883 | 54817997 | 54825977 | immunity |
| HORAC | Bone Gamma-Carboxyglutamate Protein/osteocalcin | BGLAP | 1 | ENSOARG00000005620 | 104826771 | 104827743 | bone formation |
| RRAD And GEM Like GTPase 1 | REM1 | 13 | ENSOARG00000000783 | 60377529 | 60386368 | Muscle development |
| BCL2 Like 1 | BCL2L1 | 13 | ENSOARG00000001262 | 60531757 | 60581987 | apoptosis in the immune system, kidney, and bone cells |
| Aurora Kinase A | AURKA | 13 | ENSOARG00000018369 | 58840124 | 58858898 | cell division |
| Solute Carrier Family 7 Member 11 | SLC7A11 | 17 | ENSOARG00000014253 | 19076299 | 19158878 | coat color |
| Abhydrolase Domain Containing 5, Lysophosphatidic Acid | ABHD5 | 19 | ENSOARG00000004222 | 15488893 | 15526261 | Milk yield |
| Signal Transducer and Activator Of Transcription 1 | STAT1 | 2 | ENSOARG00000013903 | 192154022 | 192352057 | Immunity during early pregnancy |
| Integrin Subunit Alpha V | ITGAV | 2 | ENSOARG00000016607 | 121567325 | 121672421 | reproduction |
| Integral Membrane Protein 2C | ITM2C | 2 | ENSOARG00000020673 | 231740173 | 231752466 | Immunity |
| Tyrosinase | TYR | 21 | ENSOARG00000003888 | 5744320 | 5858941 | Pigmentation |
| NADH:Ubiquinone Oxidoreductase Subunit B8 | NDUFB8 | 22 | ENSOARG00000015378 | 20557569 | 20562826 | Respiration |
| Arginine Vasopressin Receptor 1A | AVPR1A | 3 | ENSOARG00000004113 | 156234256 | 156237118 | vasoconstriction of blood vessels and glycogenolysis |
|  | Chromobox 5 | CBX5 | 3 | ENSOARG00000016251 | 132139439 | 132149313 | Skeletal and bone development |
| Homeobox C6 | HOXC6 | 3 | ENSOARG00000016296 | 132338094 | 132340389 | Cardiovascular functions |
| ATP Synthase Membrane Subunit C Locus 2 | ATP5MC2 | 3 | ENSOARG00000016393 | 132715794 | 132719269 | Neurological functions in humans |
| Insulin Like Growth Factor Binding Protein 6 | IGFBP6 | 3 | ENSOARG00000016699 | 133213326 | 133216912 | Reproduction- uterine development |
| TIMP Metallopeptidase Inhibitor 3 | TIMP3 | 3 | ENSOARG00000018210 | 176462578 | 176519533 | Follicular development |
| Intraflagellar Transport 27 | IFT27 | 3 | ENSOARG00000018994 | 179967963 | 179990650 | Follicular development |
| Interleukin 1 Beta | IL1B | 3 | ENSOARG00000020866 | 60023514 | 60034494 | Immunity |
| Interleukin 1 Alpha | IL1A | 3 | ENSOARG00000020877 | 60078287 | 60088697 | Immunity |
| Casein Alpha S1 | CSN1S1 | 6 | ENSOARG00000010276 | 85089487 | 85102981 | milk quality |
| Casein Beta | CSN2 | 6 | ENSOARG00000010477 | 85116827 | 85122776 | milk quality |
| Casein Alpha S2 | CSN1S2 | 6 | ENSOARG00000010683 | 85184160 | 85195356 | milk fatty acid composition |
| Casein Kappa | CSN3 | 6 | ENSOARG00000011084 | 85309552 | 85316834 | milk proteins |
| SRA Stem-Loop Interacting RNA Binding Protein | SLIRP | 7 | ENSOARG00000002642 | 85795918 | 85801678 | sperm motility |
| Tropomyosin 1 | TPM1 | 7 | ENSOARG00000020797 | 44019267 | 44048143 | Associated with children deformities |
